# Real Time Imaging of Human Progenitor Neurogenesis

**DOI:** 10.1371/journal.pone.0013187

**Published:** 2010-10-07

**Authors:** Thomas M. Keenan, Aaron D. Nelson, Jeffrey R. Grinager, Jarett C. Thelen, Clive N. Svendsen

**Affiliations:** 1 Department of Neurology, University of Wisconsin-Madison, Madison, Wisconsin, United States of America; 2 Cedars-Sinai Regenerative Medicine Institute, Los Angeles, California, United States of America; Tokyo Medical and Dental University, Japan

## Abstract

Human neural progenitors are increasingly being employed in drug screens and emerging cell therapies targeted towards neurological disorders where neurogenesis is thought to play a key role including developmental disorders, Alzheimer’s disease, and depression. Key to the success of these applications is understanding the mechanisms by which neurons arise. Our understanding of development can provide some guidance but since little is known about the specifics of human neural development and the requirement that cultures be expanded *in vitro* prior to use, it is unclear whether neural progenitors obey the same developmental mechanisms that exist *in vivo*. In previous studies we have shown that progenitors derived from fetal cortex can be cultured for many weeks *in vitro* as undifferentiated neurospheres and then induced to undergo neurogenesis by removing mitogens and exposing them to supportive substrates. Here we use live time lapse imaging and immunocytochemical analysis to show that neural progenitors use developmental mechanisms to generate neurons. Cells with morphologies and marker profiles consistent with radial glia and recently described outer radial glia divide asymmetrically and symmetrically to generate multipolar intermediate progenitors, a portion of which express ASCL1. These multipolar intermediate progenitors subsequently divide symmetrically to produce CTIP2^+^ neurons. This 3-cell neurogenic scheme echoes observations in rodents *in vivo* and in human fetal slice cultures *in vitro*, providing evidence that hNPCs represent a renewable and robust *in vitro* assay system to explore mechanisms of human neurogenesis without the continual need for fresh primary human fetal tissue. Knowledge provided by this and future explorations of human neural progenitor neurogenesis will help maximize the safety and efficacy of new stem cell therapies by providing an understanding of how to generate physiologically-relevant cell types that maintain their identities when placed in diagnostic or transplantation environments.

## Introduction

Stem cell research promises to revolutionize our ability to treat or cure neurological disorders. After nearly two decades since neural progenitors were first discovered in mice [1] they have just begun finding application in screening for new, more effective drugs and as cell therapies for neurological disorders in humans. Critical to the success of these and future stem cell applications is understanding how the cells proliferate and differentiate into cell types of diagnostic or therapeutic importance. If developmental mechanisms are employed then our existing knowledge of human neural development becomes a powerful tool in maximizing the safety and efficacy of stem cell technologies, with the added benefit of providing basic scientists with an *in vitro* model for human neural development. If the proliferation and differentiation programs differ from those observed *in vivo* it is essential that we understand where those deviations occur so that physiologically-relevant cell types can be consistently generated and maintained, especially when transplanted into the more complex environments found *in vivo*.

We have previously shown that human neural progenitor cells (hNPCs) isolated from 8–13 week human fetal cortex can be expanded in culture for 50–60 weeks with over 150 population doublings [2]. Although hNPCs are clearly an artificial culture system, they appear to have some degree of innate programming that guides their differentiation as early passage hNPCs produce a majority of neurons when dissociated and plated while later passage hNPCs produce an increasing percentage of glia [3]. When allowed to differentiate in serum-free media over 7 days the majority of late-passage hNPCs produce GABAergic neurons and astrocytes, but not large projection neurons or oligodendrocytes [4]. BrdU incorporation levels during this period indicate that a substantial amount of proliferation continues during the differentiation process even though mitogens have been withdrawn from the culture media [5]. A subset of these divisions are attributable to neurogenesis which occurs not through direct hNPC differentiation but rather through an additional round of hNPC division [5] using a process that is influenced by basic fibroblast growth factor [6].

However, the mechanism by which hNPCs generate neurons is not well understood. One could look to our knowledge of human neural development for insight, but due to obvious ethical boundaries that restrict direct human study the specifics of *in vivo* human neurogenesis remain unclear. From studies in rodents and non-human primates we know that developmental neurogenesis occurs through a carefully orchestrated program of cell division by cells near the ventricular surface of the brain. Beginning about what would be 28 days post-conception in the human the neuroepithelial cells that constitute the primitive brain differentiate into highly-polarized, astroglial-like stem cells called radial glia (RG) [7], [8]. RGs begin to divide symmetrically and laterally along the ventricular surface, generating two new RGs per division. This phase of RG division greatly expands the surface area of the cell layer lining the presumptive cerebral vesicle [9] and ultimately controls the total number of neurons in the mature cortex [10].

At approximately 40 days post-conception RGs begin dividing asymmetrically to generate the first neurons [10] including Cajal-Retzius cells, which form the most superficial layer of the cortex called the marginal zone. Throughout neurogenesis RGs maintain processes anchored in the ventricular and pial surfaces allowing them to span the entire cortical wall. All newly born neurons use the process that extends towards the pial surface, known as the basal process, to migrate past existing cortical layers and populate the layer just below the marginal zone [11]. The layers of the cortex are thus established in an inside-out order with the oldest neurons being the deepest in the cortex. As neurogenesis proceeds other types of progenitors provide secondary sources of neurons (reviewed in [12], [13], [14], [15]). Intermediate progenitor cells (IPCs), which are also derived from RGs [16], [17], [18], migrate to the subventricular zone where they subsequently divide symmetrically to produce additional IPCs or two neurons [18], [19]. In a recent study performed in human fetal slice cultures [20], neurons also resulted from the symmetric division of IPCs derived from RG-like cells in the outer subventricular zone (OSVZ). Unlike ventricular zone RGs (vzRGs), these OSVZ radial glia (oRGs) do not possess apical processes that are attached to the ventricular surface but they do generate neurons using a self-renewing intermediate progenitor. Neurons may also be generated by the symmetrical division of an apically-bound cell type called short neural precursors (SNP) [21], [22]. Although the origin of SNPs is not yet clear they express the IPC marker Tbr2 [22] and like SVZ-localizing IPCs may be derived from RGs. As development progresses and neurogenesis wanes, RGs transition back to symmetrical division producing glia at the expense of neurons. They eventually withdraw their processes and differentiate into glia, some of which may comprise a stable progenitor pool capable of generating neurons and glia through adulthood [15].

We wanted to establish if hNPCs employ developmental mechanisms using cell types analogous to the RGs, oRGs, SNPs, or IPCs found *in vivo*. Evidence that hNPCs continue to use developmental mechanisms after many weeks of *in vitro* culture would demonstrate that hNPCs provide a relevant *in vitro* model for exploring aspects of human neurogenesis that minimizes the need for primary human fetal tissue. It could also prove useful in maximizing the safety and efficacy of emerging stem cell drugs and therapies. Here we demonstrate through time-lapse microscopy and immunocytochemical analysis that late-passage hNPCs indeed follow a 3-cell neurogenic system analogous to that observed *in vivo* in which cells with morphologies and marker profiles consistent with RGs and oRGs divide to produce an intermediate cell type that in turn divides symmetrically to produce two neuroblasts.

## Materials and Methods

### Human Neural Progenitor Cultures

Human embryonic tissue was collected from the Birth Defects Laboratory at the University of Washington, Seattle. The method of tissue collection followed the guidelines set forth by the National Institutes of Health with full IRB approval from the University of Wisconsin and the University of Washington. Experiments were performed using hNPCs from two fetal cortex samples (89 and 94 days gestation). For each sample the entire cortex was harvested, minced, and allowed to reform into neurospheres in the presence of DMEM/Ham’s F12 (7∶3), 2% B27 supplement, 20 ng/mL EGF, 20 ng/mL FGF2, 5 µg/mL heparin, and 1% penicillin/streptomycin/amphotericin B (PSA). Cells were passaged using a previously reported method [23] in which neurospheres are quartered on a McIlwain tissue chopper (Mickle Laboratory Engineering, Surrey, England). After 4 weeks in culture hNPC neurospheres were switched to growth media (DMEM/Ham’s F12 (7∶3) containing 1% N2 Supplement, 20 ng/mL EGF, 10 ng/mL leukemia inhibitory factor (LIF), and 1% PSA. No human subjects were used in this study.

### Lentiviral Infection of hNPC Cultures

Vesicular stomatitis virus G (VSV-G)-pseudotyped lentiviruses were produced by transient calcium phosphate co-transfection of 293T cells as described previously [24]. Lentiviral vector concentrations were initially normalized according to the p24 (HIV-1 capsid protein) content of supernatants measured by enzyme-linked immunosorbent assay. Ten-times concentrated stocks of lentiviruses were used for cell infection. Neurospheres were collected 5 days post-passaging and allowed to settle by gravity. Media was removed and spheres resuspended in one mL Accutase per 10 million cells, mixed gently and incubated at 37°C for 10 min. Accutase was replaced with an equal volume of 0.2% trypsin inhibitor (Sigma) and the cells were washed three times in 10 mL media. Cells were dissociated by trituration, counted on a hemocytometer, and suspended in conditioned media at 1000 cells/µL. 300,000 cells were plated per well of a 24-well plate (minimum 10 wells) and mixed with virus diluted to the desired titer in 100 mL of fresh media. Cells were exposed to a viral concentration of 75 ng p24/mL and allowed to re-associate in the presence of virus. Spheres were collected 24–72 hours later and seeded into flasks at a density of 5×10^5^ cells/mL.

### GFP Expressing Neurospheres

Lenti-GFP infected hNPC neurospheres and passage-matched, non-infected hNPC neurospheres were enzymatically dissociated in Accutase™ (10 min, 37°C) and then mechanically dissociated with fire-polished, glass Pasteur pipettes. Lenti-GFP hNPCs were mixed 1∶500 with non-infected counterparts and allowed to reform neurospheres. Mixed neurospheres were passaged weekly for at least two weeks prior to time-lapse imaging.

### Dissociated Culture Time-lapse Microscopy

To evaluate the behavior of individual cells neurospheres were dissociated into a single cell suspension using Accutase™ and seeded at ∼93,000 cells/cm^2^ in 8-well LabTek culture slides (Fisher Scientific) previously coated with laminin (100 µg/mL; 30 minutes at 37°C). Cells were allowed to plate in the wells for a minimum of 2 hours in the presence of DMEM/Ham’s F12, 2% PSA and 2% B27 supplement (Invitrogen, Carlsbad, CA) at 37°C in 5% CO_2_. A sterile 0.4 µm filter and a gas tube were then attached to the LabTek culture slide lid. The slide was transferred to the programmable stage of a Nikon TE100 inverted microscope housed within a 37°C temperature control chamber. Compressed 5% CO_2_/air gas was perfused through the gas tube to maintain constant media pH. Metamorph™ 5.0 (Molecular Devices, Downingtown, PA) was used to capture images at 3 minute intervals over 3 days.

### Neurosphere Culture Time-lapse Microscopy

Two-well LabTek culture slides (Fisher Scientific) were coated with poly-L-lysine (100 µg/mL; 60 min, room temperature) and laminin (100 µg/mL; 60 min, 37°C). Mixed Lenti-GFP:wildtype hNPC neurospheres were seeded in the chambers and allowed to attach for 60 minutes in a 37°C, 5% CO_2_ incubator. The culture slides were then transferred to the aforementioned microscope housed in a temperature control chamber. Instead of supplying gas directly to the LabTek culture slide, the slide was placed inside a smaller acrylic box fed with humidified, 5% CO_2_/95% air. Green hNPCs within each neurosphere were brought into focus and fluorescence images were acquired every 3 minutes for 2 or more days using Metamorph™ 5.0.

### Image Analysis

Acquired images were assembled into movies using Metamorph™ 5.0. The movies were used to evaluate the morphology, behavior, migration rates, and cell division symmetry of individual hNPCs and their progeny. The shape and size of each cell was quantified using the Metamorph™ 5.0 Morphometric Analysis Tools. For 3D somal area quantification mother cell areas were measured 15 minutes prior to any signs of process retraction or cell rounding that precedes cell division. Daughter cell areas were measured 3 hours after cell division.

### Immunocytochemistry

Cells were fixed in 4% PFA for 20 minutes at room temperature. Cells were permeabilized with 0.2% Triton X-100 solution in 10% serum for 35 minutes at room temperature. Cells were stained with the following primary antibodies for 1 hour at room temperature or overnight at 4°C: Nestin (Millipore, Billerica, MA), GFAP (Dako, Carpinteria, CA), GFAPδ (Abcam, Cambridge, MA), GFAPc19 (Santa Cruz Biotechnology, Santa Cruz, CA), vimentin (Developmental Studies Hybridoma Bank, Iowa City, IA), S100 (Abcam, Cambridge, MA), 4A4 (MBL International, Woburn, MA), 3CB2 (Developmental Studies Hybridoma Bank, Iowa City, IA), Tbr2 (Abcam, Cambridge, MA), Neurogenin-2 (Millipore, Billerica, MA), ASCL1 (CosmoBio, Tokyo, Japan), TuJ1 (Sigma, St. Louis, MO),and CTIP2 (Abcam, Cambridge, MA). Cells were rinsed in PBS and stained with fluorophore-tagged secondary antibodies for 35–60 minutes at room temperature. Nuclear labeling was performed using Hoechst 33258 (0.5 µg/mL in 1xPBS, Sigma, St. Louis, MO).

### Cell Analysis

Cell identities were determined based on morphology, migration speed, and marker protein expression profiles. In cell division experiments cells with morphologies consistent with those delineated using immunocytochemistry were assumed to be the same cell type. Symmetric and asymmetric divisions were classified based on the morphologies and migration behaviors of the daughter cells.

### Statistical Analysis

Cellular data was evaluated statistically using either the Student’s t-test or One-way ANOVA with either the Neuman-Keuls or Tukey post test. Statistical significance was held at P<0.05. Values are plotted as the mean ± SEM. Statistical analyses were performed using Graphpad Prism® Software (Graphpad Software Inc, San Diego, CA).

## Results

### Dissociated hNPCs Display Three Distinct Cell Morphologies

**Figure 1:**
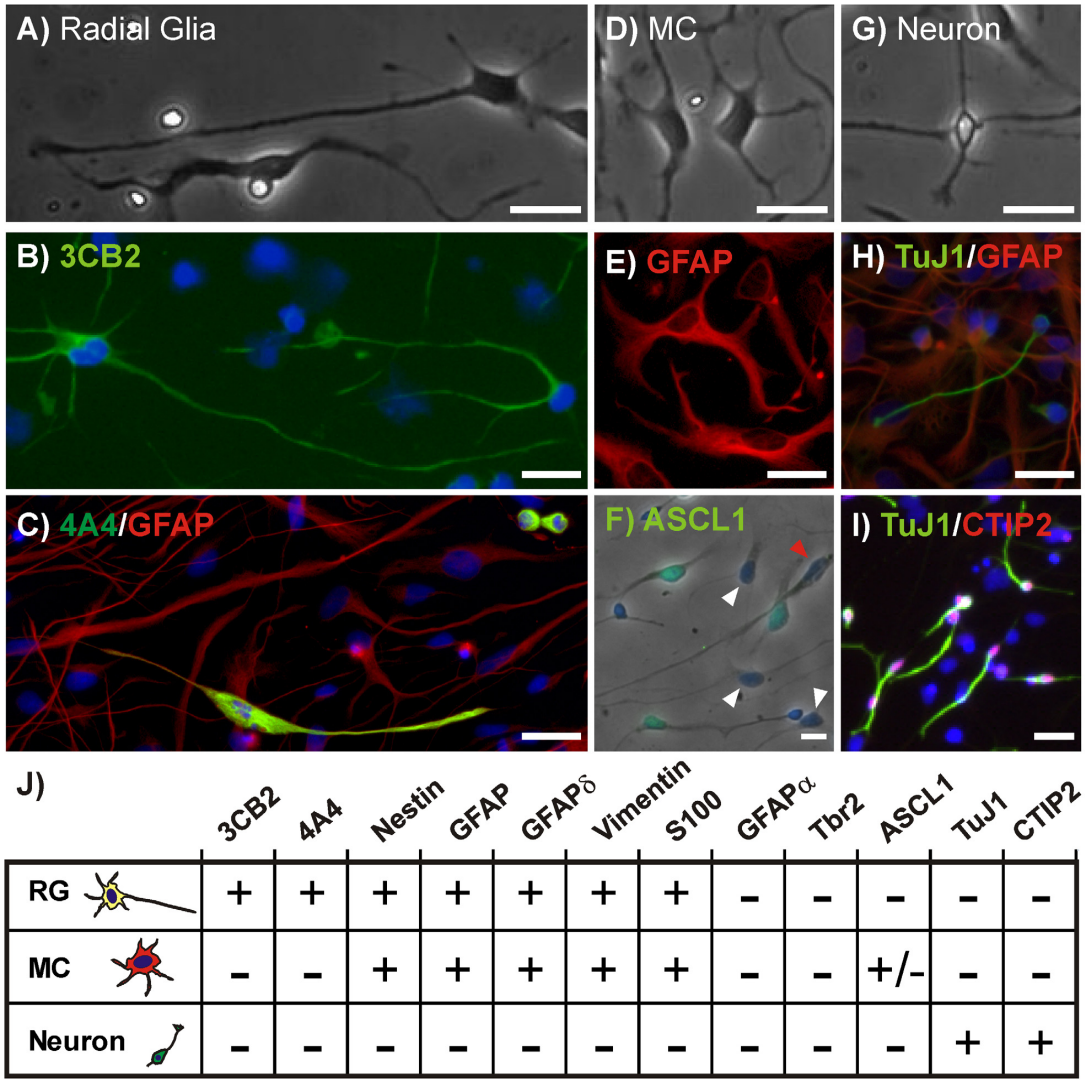
Three Distinct hNPC Morphologies and Marker Profiles. hNPC-derived radial glia (RG) displayed radial morphology with processes extending over 100 um (**A**) and expressed the radial glial markers 3CB2 (**B**), 4A4 and GFAP (**C**). Multi-polar cells (MC) (**D**) stained positively for GFAP (**E**) and the IPC marker ASCL1 (**F**). ASCL1 was not expressed in all MCs (white arrowheads) nor in any of the RGs (red arrowhead). Neurons (**G**) stained positively for TuJ1 (**H**) and CTIP2 (**I**). Scale bar  = 25 um. (**J**) Marker profiles for hNPC cell types. All images are representative examples of the immunocytochemistry performed for each marker protein.

Because ∼20 weeks is the length of time it takes to expand primary cortical neural progenitors into hNPC cultures sufficiently large for large-scale *in vitro* screening or transplantation applications (∼4×10^9^ cells), we examined the nature of hNPCs cultured for 20 or more weeks *in vitro*. At this stage the cultures give rise to approximately 20% neurons upon differentiation for 7 days [2], [25]. We found that when these late-passage hNPCs were dissociated and plated on laminin-coated glass in the absence of growth factors three morphologically-distinct cell types were consistently generated. The first cell type (Fig. 1A) had a multipolar morphology characterized by either a single long, projecting process or by two opposed, projecting processes many exceeding 100 µm in length. This cell type was non-migratory and expressed the radial glial marker 3CB2 (Fig. 1B), which was recently shown to label specific isoforms of the intermediate filament protein vimentin [26]. During and shortly after division it also expressed a phosphorylated form of vimentin, identified by the antibody 4A4 [27] (Fig. 1C), shown to identify dividing RGs [28]. This cell type did not stain positively for the IPC markers Ngn2, Tbr2, and ASCL1 [29], the neurospecific markers TuJ1 and CTIP2, or the GFAPα isoform identified by the GFAPc19 antibody. However, the cells did express other GFAP isoforms (Fig. 1C) including GFAPδ (data not shown), as well as Nestin, vimentin, and S100 (data not shown). We will subsequently refer to this cell type as a radial glia (RG). The second cell type (Fig. 1D) had a soma similar in size to RGs but did not possess long projecting processes and thus will be referred to as a multi-polar cell (MC). In dissociated cultures MCs often adopted very spread, fibroblastic morphologies. Like RGs, MCs stained positively for GFAPs (Fig. 1E) including GFAPδ as well as vimentin, S100 and nestin. MCs did not stain positively for 3CB2, 4A4, GFAPα, Tbr2, CTIP2, or TuJ1 but did stain positively for the IPC marker ASCL1 (Fig. 1F). The final cell type was a small uni- or bi-polar cell with a soma that was much smaller than that of either RGs or MCs (Fig. 1G). This cell type migrated rapidly around the culture surface at velocities up to 10 µm/hr led by the longest or largest process. As might be expected for a post-mitotic cell type, the cells were nestin and GFAP negative and expressed high levels of the neurospecific marker TuJ1 (Fig. 1H) and the Layer 5/6 cortical marker [30] CTIP2 (Fig. 1I). We will refer to these cells as neurons.

### RGs Divide Both Symmetrically and Asymmetrically

**Figure 2:**
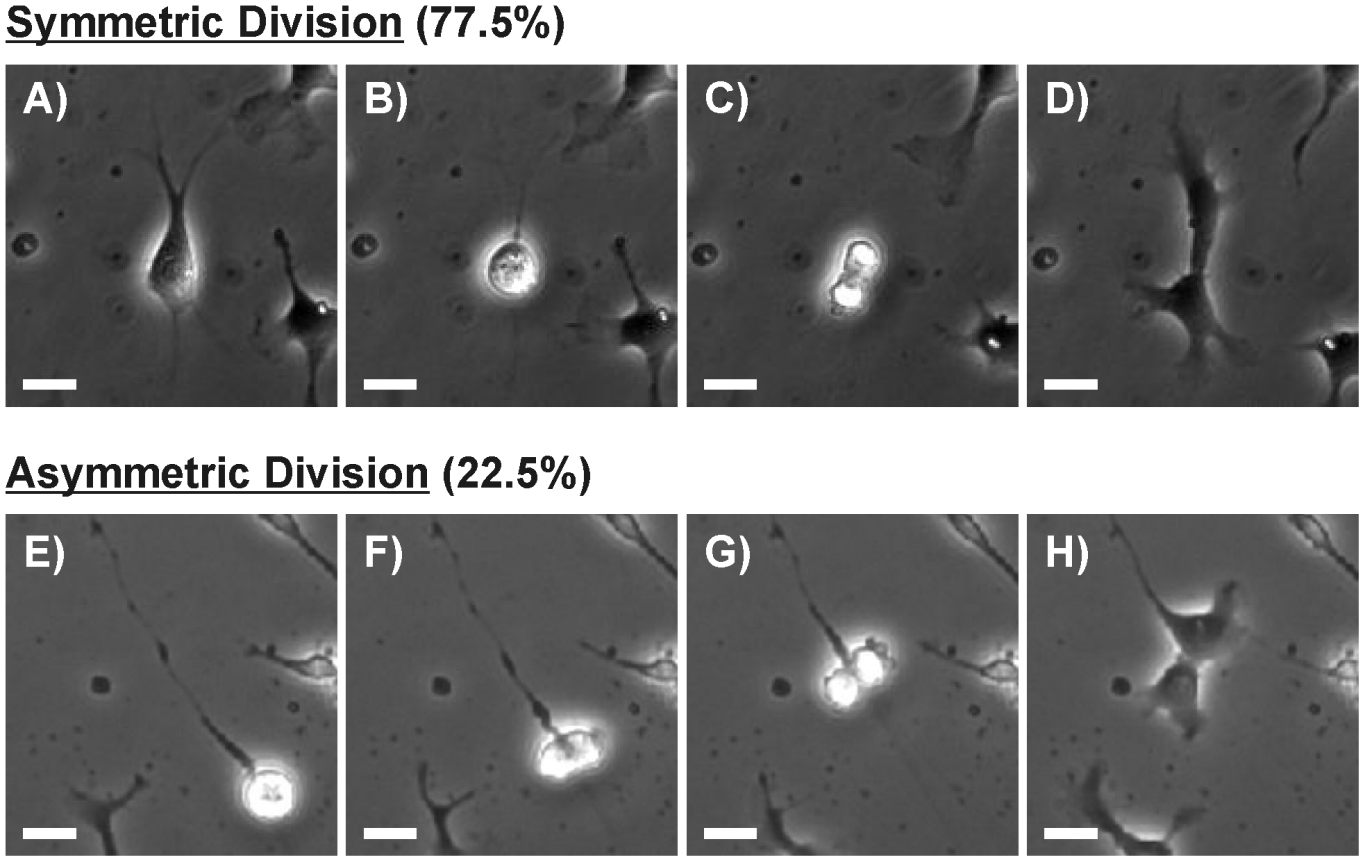
Division in Dissociated hNPC Cultures. (**A**) Phase contrast image of a symmetrically dividing hNPC which comprised 77.5% of all divisions. (**B**) The cell retracts all processes and rounds up. (**C**) Division and cytokinesis occurs on a plane perpendicular to the former major axis of the mother cell. (**D**) Daughter cells are morphologically indistinguishable from one another. (**E**) Phase contrast image of an asymmetrically dividing hNPC which comprised 22.5% of all divisions in 2D dissociated cultures. (**F**) The cell rounds up but does not fully retract its major process. (**G**) The cell divides along a plane approximately parallel to the cell’s major axis. (**H**) One daughter cell maintains the radial process while the other adopts a MC morphology. Scale bar  = 25 um.

In order to establish the types of division exhibited by these cultures we followed individual cells using time-lapse microscopy. Of the 54 dividing cells observed 77.5% underwent symmetric division and 22.5% underwent asymmetric division based on the morphology of the progeny. While both RGs and MCs underwent symmetric division, only RGs divided asymmetrically. Each type of division was defined by a unique sequence of events. A cell undergoing symmetric division (Fig. 2A) would first retract all of its processes and round up (Fig. 2B). Cytokinesis would then take place along a plane approximately perpendicular to any major axis of the cell (Fig. 2C). The cell would then divide and two equally-sized daughters with very similar morphology would be formed (Fig. 2D). In all observed RG asymmetric divisions the cell never fully retracted its processes. Instead the processes retracted only slightly and narrowed significantly just prior to cell division (Fig. 2E). The soma rounded up, but in contrast to symmetric division the division plane was approximately parallel with the major axis of the cell (Fig. 2F–H). In all cases one of the daughter cells maintained the primary process while the other adopted a MC morphology (Fig. 2H). A typical asymmetric division is shown in Movie S1 in which a RG extends a long radial process that is subsequently retained while the cell body divides. To our knowledge, this is the first report of a human RG dividing asymmetrically in dissociated culture.

### RGs in hNPC Cultures Produce Neurons Through An Intermediate Progenitor

**Figure 3:**
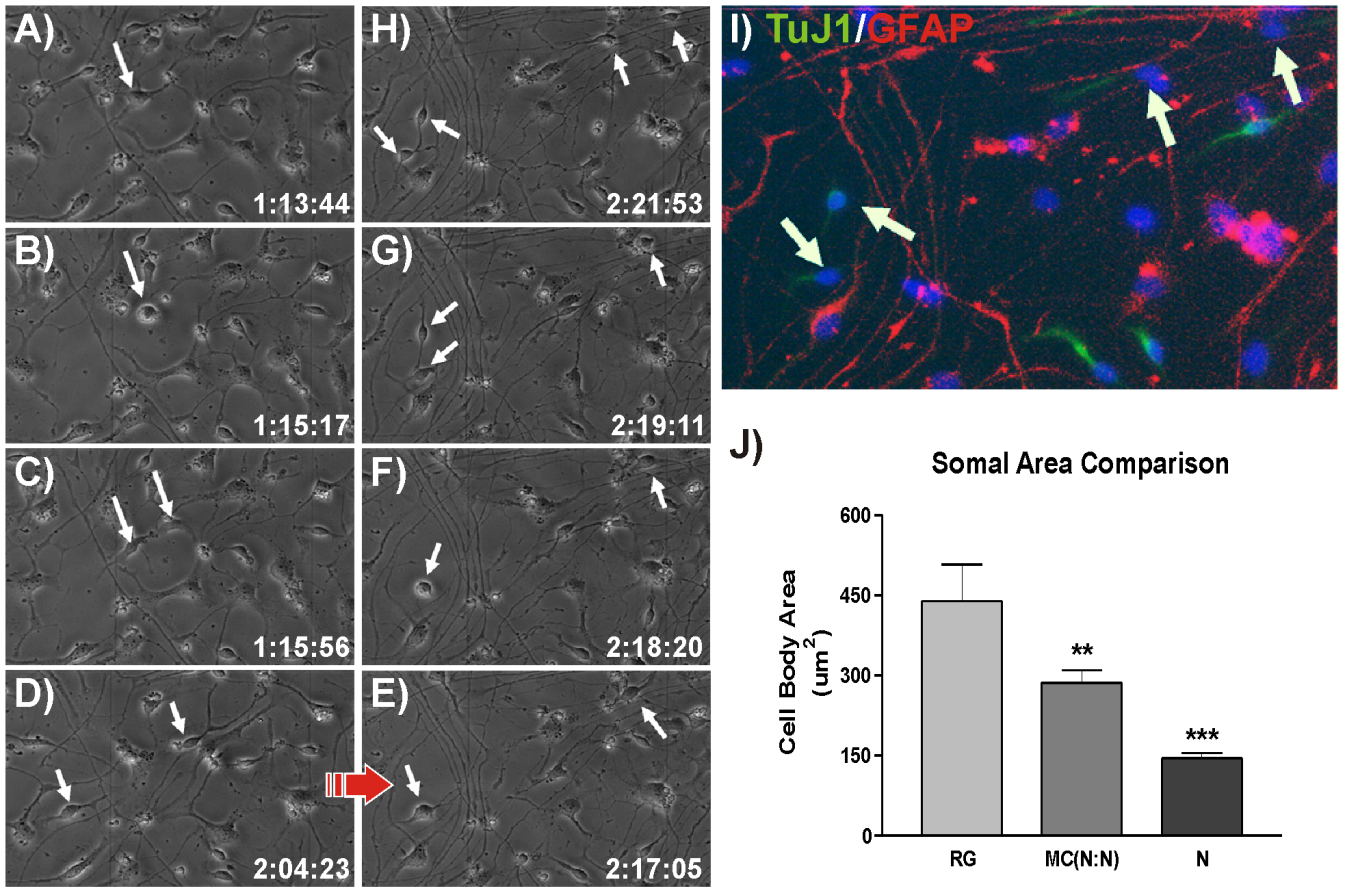
hNPC Neurogenesis Uses a 3-Cell Hierarchy. (**A–C**) Phase contrast image of a hNPC-derived RG rounding up and dividing symmetrically to produce two MCs. (**D–E**) The MCs migrate away from one another. (**G–H**) In near synchrony the daughter cells divide again to each produce two neurons that are TuJ1 positive (**I**). (**J**) A comparison of somal sizes shows that RGs are 1.5 times larger than their neurogenic multipolar daughters [MC(N:N)], which in turn are approximately twice the size of the final neuron daughters (Mean ± SEM, **p<0.01, ***p<0.001).

Although rodent RGs at early stages of development have been shown to directly give rise to neurons via asymmetric divisions *in vivo* [11], [31], at no time was a cell with neuronal morphology observed to result directly from RG division in hNPC cultures. The lack of such observations indicates that late passage hNPCs in this model follow a different neurogenic scheme. To better understand how neurons are generated we identified cells with neuronal morphology that also stained positively for TuJ1. We then used corresponding time-lapse movies to track the division history of each neuron as well as the morphology and behavior of respective parent cells. We discovered that neurons always resulted from the symmetric division of an MC, and that neurogenic MCs were the products of both symmetric and asymmetric RG divisions. Figure 3 shows a typical example of this 3-cell neurogenic hierarchy (Movie S2). A RG (Fig. 3A) retracts its processes fully (Fig. 3B) and divides symmetrically (Fig. 3C) to produce two MC daughter cells (Fig. 3D). After more than 24 hours (Fig. 3F–H) the two daughters again round up and divide symmetrically in near synchrony to produce two TuJ1-positive neurons each (Fig. 3I). Quantification of the somal areas of the three cell types in the observed neurogenic hierarchy revealed that parent RG's were ∼1.5 times the size of intermediate MCs which in turn were ∼2.0 times the size of their neuron daughters (Fig. 3J). To better ascertain the identity of the MC intermediate in the 3-cell neurogenic system we immunostained hNPC cultures for ASCL1, Neurogenin 2 (Ngn2), and Tbr2. ASCL1 is a marker of IPCs that generate GABAergic neurons [29]. Ngn2 and Tbr2 specifically label IPCs that generate glutamatergic neurons [32], [33]. We found that MCs did not express Ngn2 or Tbr2 but did express ASCL1 in 38% of the population.

Because the human brain requires orders of magnitude more neurons than those of rodents it seems plausible that hNPCs would rely to a greater degree on intermediate progenitors to amplify neuron production in lieu of direct neurogenesis via RG asymmetric division. However, we also recognize that dissociated cultures lack the cell-cell contact and growth factor-rich environment present in neurospheres or organized tissue, and the observed 3-cell hierarchy may be an artifact of dissociation or the 2D culture environment. To establish whether the 3-cell neurogenic system observed in dissociated cultures is consistent in cultures where cell-cell contact and signaling have not been disrupted we performed analogous time-lapse studies using whole hNPC neurospheres. Neurosphere cultures cannot fully recapitulate the *in vivo* cortical microenvironment, but they do provide a more supportive tissue-like environment in which hNPCs are known to thrive relative to dissociated cultures [23].

### hNPC Neurosphere Cultures Also Produce 3 Distinct Morphologies

**Figure 4:**
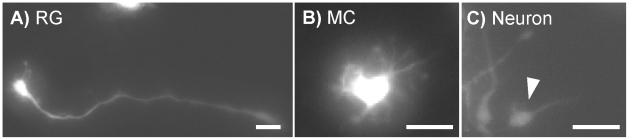
hNPC Morphologies in Neurosphere Cultures. Lenti-GFP infected hNPCs growing within neurospheres display 3 distinct morphologies consistent with RG (**A**), MC (**B**) and neuronal morphologies (**C**) found in dissociated cultures. Scale Bar  = 50 um.

To allow single cells within whole neurospheres to be visualized, we created hybrid neurospheres consisting of both Lenti-GFP infected hNPCs and passage-matched, non-infected cells mixed in a 1∶500 ratio. We have previously shown that infection of hNPC with lentiviral constructs has no deleterious effects on proliferation or differentiation [34], [35], [36]. Time-lapse fluorescence microscopy of whole spheres plated onto a laminin substrate in the absence of growth factors revealed the same three morphologically-distinct cell types as those observed in dissociated cultures. RGs had 1–2 long projecting processes that often exceeded 100 µm in length (Fig. 4A). The only notable morphological difference was RGs in neurosphere cultures had finer and more dynamic minor processes emanating from the cell body that extended and retracted more rapidly than those in dissociated cultures. MCs in neurosphere cultures were nearly indistinguishable from their dissociated culture counterparts (Fig. 4B), but like RGs had processes that were finer and more active. Neurons (Fig. 4C) had small somas with one or two short processes emanating from the cell body. The cells were highly migratory and led by the longest or largest process.

### hNPC Neurosphere Cultures Display Symmetric and Asymmetric Divisions

**Figure 5:**
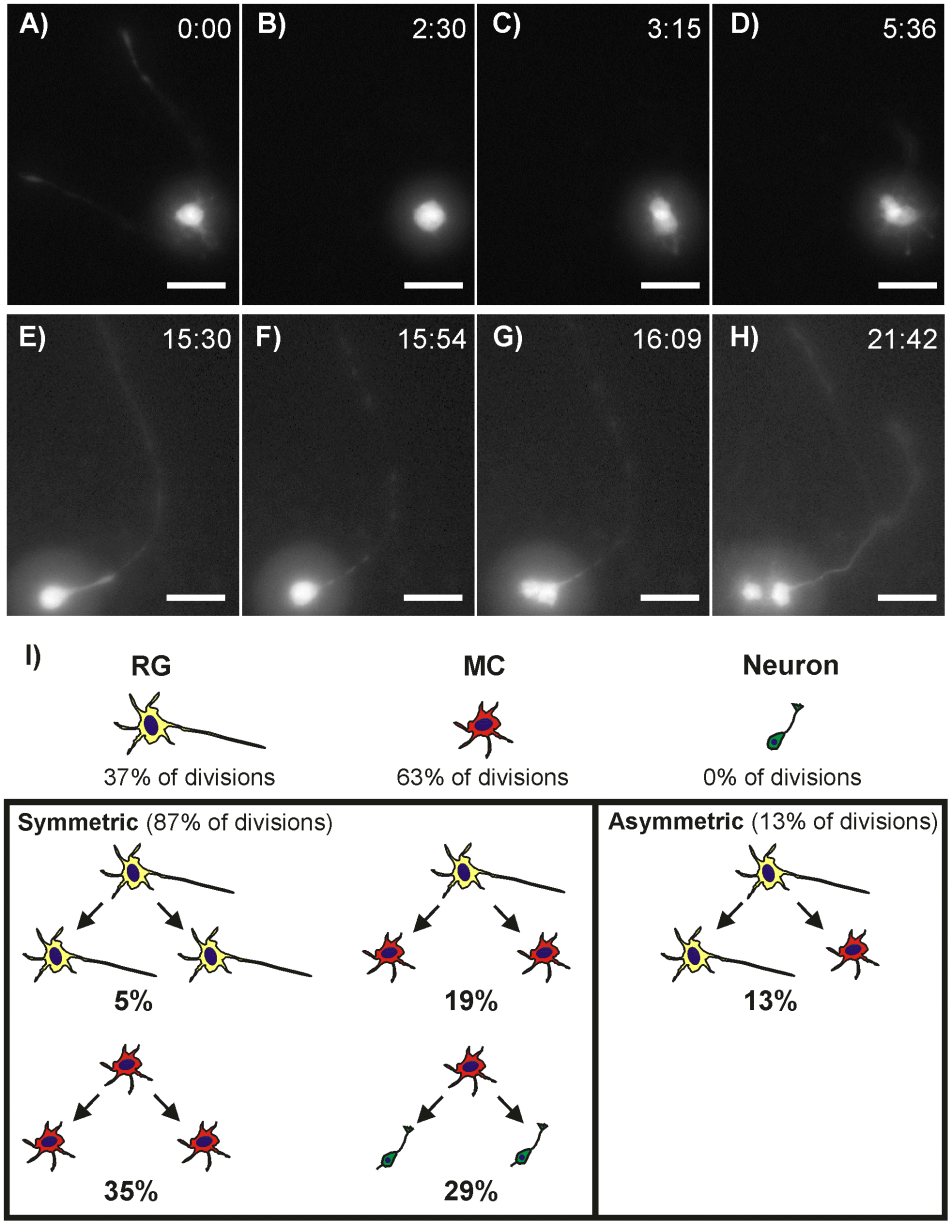
hNPC Division Patterns in Neurosphere Cultures. (**A**) A symmetrically-dividing RG completely retracts its processes (**B**) and divides symmetrically on a plane approximately perpendicular to the RG’s major axis (**C**) to produce two MCs (**D**). (**E**) An asymmetrically-dividing RG does not fully retract its process but does evacuate the process cytoplasm (**F**) and divides along a plane approximately parallel with the major axis producing a MC and RG daughter (**H**). (**I**) RG divisions constitute 37% of all divisions, MCs 63% and neurons 0%. Out of all observed divisions 87% were symmetric and 13% asymmetric. Only RGs divided asymmetrically while both RGs and MCs divided symmetrically. Neurons were only generated through the symmetric division of MCs and never as a direct result of RG division.

The divisions of 63 lenti-GFP cells within the cultured neurospheres were clearly observed using fluorescence time-lapse imaging. All divisions arose from either RGs (37%) or MCs (63%). No divisions occurred in cells morphologically similar to TuJ1-expressing neurons. As in dissociated cultures, RGs were observed to divide both symmetrically and asymmetrically while MCs only divided symmetrically. Symmetric divisions (Movie S3) were again characterized by complete retraction of all processes (Fig. 5A–B) and a division plane approximately perpendicular to any major axis of the cell (Fig. 5C). Likewise, asymmetric divisions (Movie S4) were characterized by incomplete retraction of RG processes (Fig. 5E–F) and a division plane approximately parallel to the major axis of the cell (Fig. 5G). In some cases the processes were undetectable at the moment of mitosis due to cytoplasmic evacuation that reduced the eGFP signal. However, these divisions were still deemed asymmetric based on the rapidity with which the eGFP signal returned following cytokinesis, the morphologies of the daughter cells, and the orientation of the division plane relative to the major axis of the mother cell. In neurosphere cultures, 87% of divisions were symmetric and 13% were asymmetric.

Within the RG subpopulation cells followed three different division strategies with each producing different sets of daughter cells (Fig. 5I). In the first strategy, the cell completely withdrew all long projection processes to form a spherical intermediate that then divided into 2 equally-sized cells. Both cells immediately sent out 1–2 long projection processes and adopted morphologies characteristic of RGs. These proliferative symmetric divisions comprised 13% of all RG divisions (5% of total divisions). In the second division strategy, the RG again fully retracted all projecting processes but instead of producing two RG daughters the cell produced 2 equally-sized MCs. This strategy was observed in 52% of RG cell divisions (19% of total divisions). In the third division strategy the cell only partially withdrew its projecting processes and split into one RG that maintained the parent process and one MC. This asymmetric division strategy was observed in 35% of all RG divisions (13% of total divisions). At no time did we observe a RG directly give rise to a cell with neuronal morphology through either asymmetric or symmetric division, thus confirming observations in dissociated cultures.

As previously mentioned, within the MC subpopulation we observed only symmetric cell divisions (Fig. 5I). In all cases the cell withdrew all processes to form a spherical intermediate. The cell then divided symmetrically to produce 2 daughter cells that both adopted either MC morphologies (55% of MPC divisions) or neuronal morphologies (45% of MPC divisions). Like in dissociated hNPC cultures, neurons were highly motile with a single leading process guiding their migration path. No RGs were produced from any MC division.

### Neurogenesis in hNPC Neurosphere Cultures Supports a 3-cell Hierarchy

**Figure 6:**
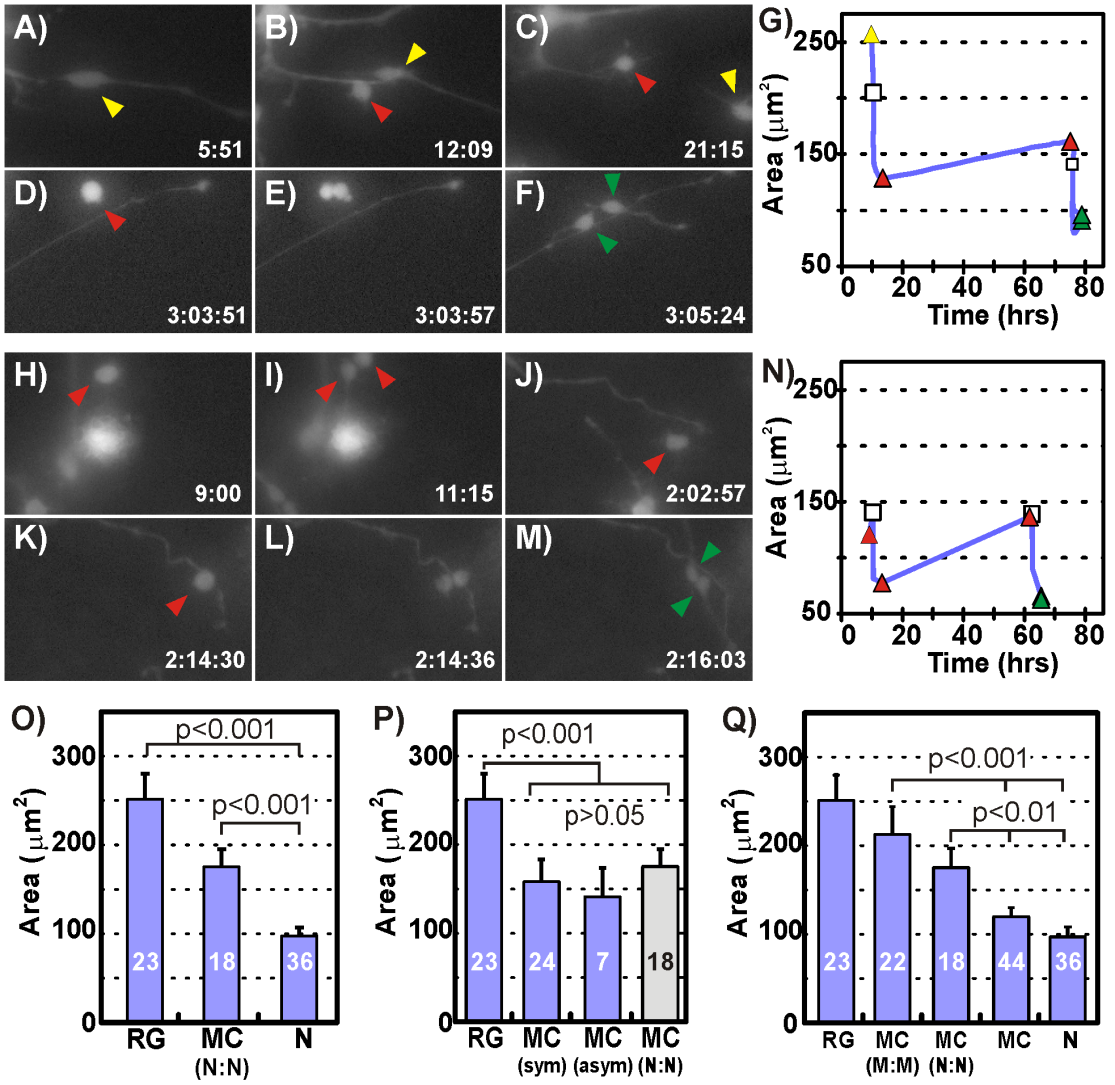
Neurosphere Cultures Utilize a 3-Cell Neurogenesis Hierarchy. Fluorescence micrographs of a lenti-GFP infected RG (**A**) dividing asymmetrically (**B**) to produce a RG (yellow arrowhead) that migrates out of the field of view and a MC (red arrowhead)(**C**). The MC divides again (**D–E**) approximately 2 days later producing highly motile neurons (green arrowheads)(**F**). (**G**) Somal size comparison for the cell sequence shown in (A–F) details the division of the RG (yellow triangle) into the MC (red triangle). The MC grows slightly over 2 days before it divides again (white square) into 2 neurons (green triangles). Fluorescence micrographs of a lenti-GFP infected MC (**H**) dividing symmetrically (**I**) to produce two more MCs one of which migrates out of the field of view (**J**). The other MC (red arrowhead) continues to grow and migrate until it divides 2 days later (**K–L**) into two neurons (**M**). (**N**) Somal size comparisons for the cells in (H–M) reveals that the daughter MC grows to the size of its mother MC before itself dividing into two neurons. (**O**) Comparison of the somal areas for all cells shows that the three cell types participating in the 3-cell hierarchy have distinct sizes. (**P**) A comparison of the somal areas of all RGs, all MCs derived from symmetric (MC_sym_) and asymmetric (MC_asym_) division, and neurogenic MCs (MC_(N:N)_) provides circumstantial evidence that all RG daughters may be neurogenic MCs. (**Q**) Inclusion of MC-genic MCs and their MC daughters in somal area comparisons and the lack of congruence with the somal areas of other cell types suggest the presence of another MC subpopulation.

To determine whether hNPCs in the supportive environment of the neurosphere utilize the same 3-cell neurogenic system observed in dissociated cultures we searched the time-lapse data for cells that underwent two successive divisions. We were able to identify two cases in which double divisions led to neuron daughters. In the first case (Movie S5) a RG (Fig. 6A) divided asymmetrically (Fig. 6B) to produce a RG daughter (yellow arrowhead) and a MC daughter (red arrowhead). The RG daughter migrated out of the field of view (Fig. 6C) but the MC daughter divided symmetrically (65 hours later) to produce two neurons (Fig. 6D–F), thus demonstrating that the 3-cell neurogenic hierarchy also exists in 3D culture environments. In agreement with values calculated for dissociated cultures, somal area comparisons revealed that the RG mother was approximately 1.6 times the size of its MC daughter, which subsequently divided into neuron daughters that were approximately half of its size (Fig. 6G).

The second double division case revealed a variation in hNPC neurogenesis. In this instance (Movie S6) a MC (Fig. 6H) divided into two MC daughters (Fig. 6I). Although one of the MC daughters migrated out of the field of view, the other subsequently divided again (Fig. 6J–L) to produce two neurons (Fig. 6M). The somal area comparison revealed that the original MC was of similar size to the MC in Fig. 6G and divided into MC daughters that were half its size. However, the MC daughter that stayed within the field of view grew back to the size of its MC mother before dividing into neurons that were half of its size (Fig. 6N). This data suggests that MCs that are morphologically indistinguishable from MCs in the 3-cell neurogenic system are capable of undergoing self-renewing symmetric divisions prior to generating neurons.

Because we could capture only one example of a double division that directly demonstrated the existence of a 3-cell neurogenic system in hNPC neurosphere cultures we decided to mine the single division data and see if it also supported a 3-cell system. We began by measuring the somal areas of all mother and daughter cells in the 63 divisions observed in hNPC neurospheres. Cells were classified into 7 groups based on morphology and division patterns: 1) RGs, 2) MC daughters generated through RG symmetric division (MC_sym_), 3) MC daughters generated via RG asymmetric division (MC_asym_), 4) MC mothers that divided into neurons (MC_(N:N)_), 5) MC mothers that divided into MCs (MC_(M:M)_), 6) MC daughters (MC), and 7) neuron daughters (N). Mother cell somal areas were measured 15 minutes prior to the first signs of process retraction or cell rounding and daughter somal areas were measured 3 hours after cell division.

We found that the somal areas of the entire RG population, regardless of whether they were mother or daughter cells, were statistically identical to that of the RG in Fig. 6A–F that gave rise to neurons using the 3-cell hierarchy (Fig. 6O). Similarly, all neurogenic MC mothers and all neuron daughters within hNPC cultures had the same respective somal areas as their 3-cell neurogenic hierarchy counterparts highlighted in Fig. 6A–G. When we compared the sizes of the MCs derived from RG symmetric and asymmetric division with neurogenic MC mothers we found that they were statistically identical in size (Fig. 6P) suggesting that they may represent the same cell population.

If the MC population were primarily self-renewing intermediate progenitors that facilitate neuron production from RGs we would expect MC daughters to be between 75–150 µm^2^ based on Fig. 6N, and most MC_(M:M)_ cells to be the same size as MC_(N:N)_ cells. The somal area of MC daughters was indeed found to be 119.7±8.1 µm^2^ but the MC_(M:M)_ cells could not be statistically distinguished from either RGs or from MC_(N:N)_. MC_(M:M)_ cells were sizably larger than the double dividing MC detailed in Fig. 6H–N suggesting that MC_(M:M)_ cells as classified in this study did not strictly encompass self-renewing IPCs but likely included significant numbers of other cell types with multi-polar morphologies.

## Discussion

*In vitro* time-lapse imaging studies have provided key insights into rodent neurogenesis from the identification and elucidation of the role IPCs play in cortical development [16], [17], [18], [37] to the construction of lineage trees from clonal neural progenitors that demonstrated the diverse population of cortical cells that result in the absence [38] and presence [39] of exogenous growth factors. However, given the significant developmental, morphological, and cytoarchitectonic differences known to exist between rodent and human brains [14], [40] it is unclear whether the mechanisms of neurogenesis thoroughly elucidated for rodent neural progenitors apply to human cells, especially fetal-derived neural progenitors expanded for 20+ weeks *in vitro*. Rodent neural progenitors clearly follow innate programs of proliferation and differentiation even in extremely artificial *in vitro* environments [38], [39], [41], but it is unknown whether the same applies to human progenitors. Here, we explored neurogenesis in fetal-derived human neural progenitors using the single cell analysis and direct lineage tracing capabilities afforded by time-lapse microscopy. To our knowledge this is the first detailed examination of neurogenic mechanisms in cultured human neural progenitors using time-lapse imaging.

In this study we showed that after more than 20 weeks in culture, hNPCs consistently generated three morphologically-distinct cell types in both dissociated and neurosphere cultures. The three cell types exhibited morphologies, marker protein expression profiles, and migration behaviors characteristic of RGs, IPCs, and neurons observed *in vivo* [11], [18], [20], [28], [29], [30]. Time-lapse imaging in both dissociated and neurosphere cultures revealed a 3-cell neurogenic system in which RGs divide symmetrically or asymmetrically to produce IPCs. IPCs then divide symmetrically to produce CTIP2^+^ neurons. Immunocytochemical analysis revealed a proportion of the IPCs to be GABAergic neuron producing IPCs and it is likely that these are the cells mediating neurogenesis from RGs. In the developing cortex CTIP2-expressing cells reside in Layer 5 and consist primarily of glutamatergic projection neurons. However, the presence of GABAergic CTIP2^+^ neurons have also been reported [30].

The similarities in marker protein expression, cell morphology, division patterns, and neurogenic scheme between hNPC cultures and *in vivo* neurogenic progenitors provide significant evidence that hNPCs utilize developmental mechanisms to generate neurons even in artificial *in vitro* environments. Further characterization including functional analysis is needed to determine if hNPC-derived RGs are most similar to vzRGs or oRGs. As previously mentioned hNPC-derived RGs did not directly generate neurons as is observed for rodent vzRGs [11], [18] indicating they may be more similar to oRGs. The hNPCs used in this study were isolated from cortices in mid-neurogenesis in which vzRGs already span the cortical thickness and neurogenesis has been active for 40 days. It is conceivable that RGs capable of directly producing neurons had already done so and progressed to neurogenesis via IPCs or were destroyed during isolation. Alternatively, the addition of mitogens and the culture techniques used may have selected for progenitors only capable of generating neurons through intermediates. Our current understanding of oRGs indicates they produce neurons either exclusively or predominantly through IPCs and given the gestational age of the tissue from which the hNPCs were derived it is likely that a large number of hNPC-derived RGs originate from OSVZ progenitor populations [14].

The identity of the 62% of MCs not expressing ASCL1 remains unclear. Glia production occurs in the later stages of cortical development and given that hNPCs are derived from 12–13 week human cortex and cultured for over 20 weeks it is possible that significant numbers of glial progenitors [15] and post-mitotic glia are present. However, we also cannot rule out the possibility that there exists a sub-population of self-renewing multipolar progenitors that generate neurons via symmetric division. IPCs have been suggested to have self-renewal capabilities [14], [22] and one could argue Fig. 6H–N illustrates one such incidence. However, an equally strong argument could be made for Fig. 6H–N to be evidence of a unique self-renewing progenitor, separate from RGs or IPCs, that directly gives rise to neurons. In support of an exclusively 3-cell system, the somal size comparisons in Fig. 6P–Q provide evidence that neurogenic MC cells are likely the same as MCs generated solely through RG division, but without more definitive markers the link cannot be confirmed.

The data we have presented suggests for the first time that human neural progenitors cultured *in vitro* utilize a robust neurogenic mechanism that capitalizes upon IPCs to facilitate and amplify neuron production as is observed in both rodent and human fetal slice culture experiments. Given that human neurogenesis is approximately 10 times longer than in rodents but many orders of magnitude more neurons separate the species, it is perhaps not surprising that human cells would employ a predominantly 3-cell neurogenic strategy. Utilization of an IPC, especially one that self-renews, greatly amplifies the number of neurons that can be generated with each RG division and would provide a way for humans with their large and expansive brains to generate and organize cortical neurons in a timely and efficient manner.

The use of developmental mechanisms by hNPCs suggests they may represent an easy-to-culture, expandable alternative to fresh human tissue in exploring certain aspects of human neurogenesis. *In vitro* hNPC cultures cannot provide the same organized cellular architecture and growth factor rich environment of living tissue and thus may have greatest impact on investigations where surrounding tissue plays a negligible or perhaps confounding role. In addition, those seeking to develop new drugs or therapies for treating specific neurological disorders [42], [43], [44], [45], [46], [47] may be able to use hNPCs in coordination with known developmental differentiation programs to generate large populations of physiologically-relevant cell types; greatly increasing the efficacy of resulting treatments.

The safety and efficacy of emerging neural stem cell applications is critically dependent upon a thorough understanding of the mechanisms by which the cells proliferate, grow, and differentiate into neurons. Although the embryonic or fetal origin of the cells suggests the use of developmental mechanisms, the fact that the cells must be cultured for long periods of time in artificial culture conditions opens the possibility for very different cell types and behaviors than those found in the developing animal. Using time-lapse imaging, direct lineage tracing and immunocytochemical analysis we have shown that human neural progenitors cultured for over 20 weeks *in vitro* continue to utilize developmental mechanisms consistent with observations in the rodent and in human slice cultures.

## Supporting Information

Movie S1 This movie shows the asymmetric division of a RG in a dissociated hNPC culture.
(13.47 MB MOV)

Movie S2 This movie shows the symmetric division of an RG which generates two intermediate progenitors that subsequently divide symmetrically to produce 2 TuJ1 positive neurons.
(20.35 MB AVI)

Movie S3 This movie shows the symmetric division of a lenti-GFP hNPC within a seeded neurosphere.
(6.58 MB MOV)

Movie S4 This movie shows the asymmetric division of a lenti-GFP hNPC within a seeded neurosphere.
(68.72 MB MOV)

Movie S5 This movie shows the 3-cell hierarchy is also used to generate neurons in hNPCs not removed from the cell-cell contact and growth factor rich environment of a neurosphere.
(39.08 MB MOV)

Movie S6 This movie shows a self-renewing division of an MC followed by the symmetric division of a daughter MC into cells with neuronal morphology.
(32.65 MB MOV)
